# *Dietary Guidelines* should reflect new understandings about adult protein needs

**DOI:** 10.1186/1743-7075-6-12

**Published:** 2009-03-13

**Authors:** Donald K Layman

**Affiliations:** 1 Department of Food Science & Human Nutrition, University of Illinois, Urbana, IL 61801, USA

## Abstract

*Dietary Guidelines for Americans *provide nutrition advice aimed at promoting healthy dietary choices for life-long health and reducing risk of chronic diseases. With the advancing age of the population, the 2010 *Dietary Guidelines *confront increasing risks for age-related problems of obesity, osteoporosis, type 2 diabetes, Metabolic Syndrome, heart disease, and sarcopenia. New research demonstrates that the meal distribution and amount of protein are important in maintaining body composition, bone health and glucose homeostasis. This editorial reviews the benefits of dietary protein for adult health, addresses omissions in current nutrition guidelines, and offers concepts for improving the *Dietary Guidelines*.

## New concepts about protein for the *Dietary Guidelines*

• **Protein is a critical part of the adult diet**

• **Protein needs are proportional to body weight; NOT energy intake**

• **Adult protein utilization is a function of intake at individual meals**

• **Most adults benefit from protein intakes above the minimum RDA**

The developing controversy about *Dietary Guidelines *for protein stems from current perceptions that protein intakes above minimum requirements have no benefit and may pose long-term health risks. These beliefs are largely based on assumptions and extrapolations with little foundation in nutrition science. Diets with increased protein have now been shown to improve adult health with benefits for treatment or prevention of obesity, osteoporosis, type 2 diabetes, Metabolic Syndrome, heart disease, and sarcopenia [1-4]. This editorial argues that we need *Dietary Guidelines *that recognize these benefits and emphasize the right amounts of protein at specific meals.

Current perceptions are that protein is an expensive nutrient with limitations in the food supply and are reinforced by outcome measures that are based on strict cost/benefit approaches to diet formulation. This concept stems from animal science goals to maximize growth with the least expensive foodstuff. Animal feeding protocols focus on providing cheap carbohydrates as the primary energy source and limiting dietary protein to a substrate role for building new proteins. These measures are based on the body's ability to capture dietary nitrogen as body protein. Such thinking translates easily to childhood nutrition where growth and nitrogen accumulation are simple outcome measurements to confirm adequate dietary protein to maintain growth within percentile standards. Even measures of protein quality are derived from growth (Protein Efficiency Ratio: PER) and nitrogen balance (Net Protein Utilization: NPU) evaluated under conditions of limited protein intake [5]. With this history, dietary guidelines for protein evolved to provide only the minimum RDA.

During the past decade a growing body of research reveals that dietary protein intakes above the RDA are beneficial in maintaining muscle function and mobility [6] and in the treatment of diseases including obesity, osteoporosis, type 2 diabetes (T2DM), Metabolic Syndrome (MetS), heart disease, and sarcopenia [1-4]. The new research establishes health benefits and provides molecular evidence of numerous metabolic outcomes associated with protein intake or amino acid metabolism that are not reflected in the traditional measure of nitrogen balance. These outcomes include cell signaling via leucine [7,8], satiety [9,10], thermogenesis [11], and glycemic control [12,13]. The dietary protein necessary to optimize each of these metabolic outcomes is not reflected in measures of nitrogen balance and is not represented within the current concept of the minimum RDA. So what is known and what is missing in current *Dietary Guidelines*?

### Current Status and Errors of Omission

Criteria for protein requirements are based on providing the minimum essential amino acids (EAA) necessary as building blocks for new protein structures [5]. The fundamental philosophy underpinning the RDA is that once substrate requirements for EAA are met then the need for protein is satisfied. Extension of this philosophy implies that any additional amino acids beyond the minimum RDA are unnecessary and have no nutritional value.

This concept of substrate adequacy is evaluated by short-term nitrogen retention. Titration of amino acids into the diet from protein-free to surfeit intakes produces an almost linear response in nitrogen balance from negative to positive. Nitrogen balance (i.e. intake = excretion) is assumed to reflect an Estimated Average Requirement (EAR ~0.66 g/kg/d)[14]. This EAR plus a safety factor is the current RDA (0.8 g/kg/d) defined as "*the minimum daily needs for protein to maintain short-term nitrogen balance in healthy people with moderate physical activity*" [14].

At the inflection point for nitrogen balance, plasma concentrations of EAA rise rapidly stimulating amino acid oxidation [5] and this is taken as confirmation that nitrogen balance provides a measure of protein efficiency. The increase in plasma amino acids is thought to represent saturation of substrate needs (i.e. charging of tRNAs) and any additional amino acids are degraded by oxidation to energy. Amino acid oxidation serves to confirm nitrogen balance as a measure of protein efficiency. Protein intakes above the inflection point in nitrogen balance or amino acid oxidation are considered to reflect inefficient utilization or even unnecessary waste. This is the cost/benefit concept where the minimum cost of dietary ingredients is balanced with the potential for changes in body size. The goal is the largest body size for the least cost. This concept is fundamentally flawed when applied to non-growing adults.

Another major flaw in the Dietary Guidelines is the failure to recognize that dietary protein needs are inversely proportional to energy intake [15]. Current guidelines present protein needs as a percentage of energy in proportion to carbohydrates and fats. For example, *MyPyramid *represents the macronutrient goals as 55% of energy from carbohydrates, 30% from fats, and 15% from protein. At high energy intakes this balance of macronutrients is adequate. A 70 kg adult with energy intake of 2500 kcal/day would achieve a daily intake of 93 g of protein which is safely above the minimum RDA requirement of 56 g/day (i.e. 0.8 g/kg). However, if energy intake is reduced for weight management or during aging recommending protein as a percentage of energy is a serious error and potentially harmful. During weight loss, total daily energy intake is often below 1400 kcal/day. If the protein goal is represented as 15% of energy intake, daily protein intake is limiting at only 52 g. Protein needs are a function of lean tissue mass and must increase as a percentage of low energy diets.

The Food and Nutrition Board recognized the potential for biological diversity and individual choice with the DRI for macronutrients and created an Acceptable Macronutrient Distribution Range (AMDR)(14). The AMDR for protein provides a minimum RDA intake of 0.8 g/kg with a range up to at least 2.5 g/kg without any identifiable Upper Limit risk. The AMDR range was unfortunately converted into percentage of energy intakes (10% to 35% of energy) to be consistent with guidelines for carbohydrates and fat. While this provides consistency for presentation of nutrient guidelines, presenting protein as a percent of energy reduces the apparent significance of dietary protein to that of a minor energy source. This is a critical conceptual issue for *Dietary Guidelines*. Consumers must understand that absolute protein requirements (grams per day) relate to body weight and remain virtually constant across all energy intakes. If protein recommendations are maintained as an indirect relationship with energy intake (10% to 35% of energy), then *Dietary Guidelines *must emphasize that protein needs increase by approximately 1% for every 100 kcal decrease in energy intake below 2000 kcal/day.

Another error of omission in the *Dietary Guidelines *relates to recognition that the efficiency of protein utilization decreases throughout adult life [6]. During aging, there is an increase in the requirement for EAA to produce a positive response in muscle protein synthesis [16,17]. The need for total protein may not change, but the effectiveness of amino acids to stimulate muscle (and probably bone) protein metabolism decreases requiring either more total protein or greater nutrient density of EAA/total protein (i.e. protein quality). The change in efficiency of EAA use appears to be associated with the loss of anabolic drive for development of lean tissue [18]. During growth, the body has a high metabolic priority for structural development of muscle and bone driven by anabolic hormones including insulin, growth hormone, IGF-1 and steroid hormones. Further, physical activity has a positive effect on the efficiency of use of amino acids [19]. Muscle protein synthesis is stimulated by stretching and resistance activity. The converse is also true; a sedentary lifestyle reduces the efficiency of EAA use. After approximately age 30 y, the anabolic drive is lost; basal levels of hormones become largely ineffective in stimulating protein synthesis in structural tissues; and diet quality and physical activity become the limiting factors for maintaining optimal protein turnover for repair, remodeling, and recovery.

In summary, omissions in current understanding of dietary protein needs are that 1) nitrogen balance and amino acid oxidation are only useful for defining minimum protein requirements and not optimum amino acid needs, 2) protein requirement is proportional to body weight and inversely proportional to energy intake, and 3) adults need more EAA than children to maintain the efficiency of protein turnover in structural tissues.

### New knowledge about protein

Protein and amino acids contribute to multiple metabolic roles beyond simple substrates for protein synthesis. Dietary protein influences cell signaling, satiety, thermogenesis and glycemic regulations and each of these roles is initiated by increases in plasma and intracellular amino acid concentrations. These metabolic outcomes only become important with intakes above the minimum RDA. Using current measures of nitrogen balance and amino acid oxidation as the only criteria for protein requirements, these metabolic outcomes are rejected out-of-hand as inefficient and wasteful. A more logical view is that these new metabolic outcomes provide the basis for the AMDR and provide for individualization of dietary choice. Individuals can design healthy and adequate diets around the minimum RDA to prevent deficiency or design diets around higher levels of protein with additional health benefits.

**Figure 1:**
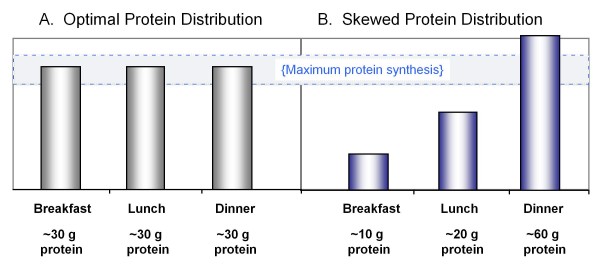
**Protein distribution at meals.** A) Ingestion of 90 grams of protein, distributed evenly at 3 meals. B) Ingestion of 90 grams of proteins unevenly distributed throughout the day. Stimulating muscle protein synthesis to a maximal extent during the meals shown in Figure 1A is more likely to provide a greater 24 hour protein anabolic response than the unequal protein distribution in Figure 1B. (Adapted from Paddon-Jones & Rassmussen Curr Opin Clin Nutr Metab Care 2009, 12: 86–90.)

Mechanisms for these metabolic outcomes are being unraveled and the effects appear to relate to the protein at each meal [20,21]. Current dietary guidelines focused on the RDA minimize the importance of protein as a central part of every meal and produce meal patterns with over 65% of protein consumed in a single large meal after 6:30 pm [22]. Most adults consume less than 10 g of protein at breakfast [23,24] (Figure 1). In children and young adults, uneven meal distribution of protein appears not to adversely affect growth. The anabolic drive maintains high efficiency of protein use for nitrogen retention even when daily protein is consumed as a single large meal. However in older adults, the quantity and quality of protein at individual meals is important. Adults require a minimum of 15 g of EAA or at least 30 g of total protein to fully stimulate skeletal muscle protein synthesis [21,25]. This response appears to be determined by the EAA leucine which serves as a critical signal for triggering initiation of muscle protein synthesis. Leucine has been well characterized as a unique regulator of the insulin-mTOR signal pathway controlling synthesis of muscle proteins [7,8]. In children and young adults, this signal pathway is regulated by insulin and dietary energy while leucine regulates the pathway in adults [26]. Current dietary patterns that provide adequate protein or leucine at only one meal produce an anabolic response only after that meal (Figure 1). This is a critical factor for protection of lean tissues during weight loss or to prevent age-related sarcopenia and osteoporosis.

The meal content of protein is also a key factor for satiety and appetite regulation [9,10]. Protein has greater satiety value than either carbohydrates or fats and reduces food intake at subsequent meals [27]. Studies of energy regulation for weight management show that replacing carbohydrates with protein reduces daily energy intake by ~200 kcal [9]. The mechanism for this satiety effect may be mediated by intestinal hormones or by reducing peak post-prandial insulin response. While the mechanism remains to be elucidated, it is clear that the improved satiety response requires >30 g of protein at a meal and that breakfast has the greatest impact on total daily energy intake [27]. As with protein turnover in muscle and bone, limiting protein intake to a single large meal late in the day reduces the satiety benefits of dietary protein [22].

The most unequivocal evidence for the benefit of increased dietary protein is derived from studies of weight management [1,28,29]. Diets with increased protein have been shown to be highly beneficial during weight loss because of their ability to correct body composition and increase satiety and thermogenesis. Higher protein diets increase loss of body weight and body fat and attenuate loss of lean tissue when compared with commonly recommended high carbohydrate low fat low protein diets [28,30]. Clearly, the major factors accounting for weight loss are the magnitude of energy restriction and individual compliance. Any diet can produce weight loss. However, long-term success with weight loss relates to maintenance of metabolically active lean tissues and research has proven that higher protein diets protect muscle and bone during weight loss. Use of conventional high carbohydrate, low fat, low protein diets results in 30% to 40% loss of lean tissue mass. Use of higher protein diets reduces lean tissue loss to <15% and when combined with exercise can halt loss of lean tissue during weight loss [30-32]. Studies also show that moderate protein diets have better long-term compliance.

The effects of protein for maintaining lean tissues appear to translate into health benefits during aging where progressive loss of structural strength and mobility are critical factors. Osteoporosis and sarcopenia have emerged as major issues during aging [2,3]. Prevention of osteoporosis is associated with physical activity and dietary calcium and protein [3]. The efficacy of calcium and protein are interrelated [3]. Calcium supplements are largely ineffective for remodeling of bone matrix if protein is limiting. Positive effects of calcium appear to require intakes of protein >1.2 g/kg to have beneficial effects. The long-held belief that increased dietary protein could cause bone loss as reflected in increase urinary calcium is incorrect [33] and protein is now recognized to increase intestinal calcium absorption in addition to enhancing bone matrix turnover [34].

Similar results have been observed with studies of muscle health in elderly where the efficiency of EAA use is reduced [16,17]. The level of EAA required to stimulate muscle protein synthesis is increased in part due to reduced anabolic stimulus of hormones. Here again it is important to distinguish the difference between outcome measures of muscle protein metabolism versus nitrogen balance. Long-term prospective outcomes with protein supplementation and muscle function are not available. However cross-sectional studies support the idea that elderly in higher percentiles of protein intake have less age-related decline in lean tissue mass [35].

Emerging health concerns relate to macronutrient choices for T2DM and MetS [4]. These conditions are characterized by dysregulation of glucose metabolism and have raised new questions about the quantity and quality of carbohydrates in the diet. Extensive research about types of carbohydrates and glycemic index have emerged but evidence is convincing that reduction in total dietary carbohydrates to less than 40% of total energy is the most effective way to improve glycemic regulations in T2DM and MetS [4].

Early research with MetS evaluated reducing dietary carbohydrates with fats [36]. While increasing dietary fats improved glycemic control and reduced cardiovascular disease (CVD) risk, the prospect of increasing dietary fat remains controversial. Replacement of carbohydrates with protein improves glycemic control measured as reduced post-prandial hyperinsulinemia [37] and in T2DM corrects hyperglycemia and HbA1c [13]. Equally important, reduced carbohydrate diets have decreased TAG, increased HDL and increased LDL particle size (i.e. LDL-C/ApoB) improving the dyslipidemia commonly associated with T2DM and MetS [4]. These conditions are 4-times more important for heart disease and all cause mortality than elevated cholesterol or LDL concentration [38].

## New understandings about protein for the *Dietary Guidelines*

### • Protein is a critical part of the adult diet

Protein should be a central part of a complete diet for adults. While physical growth occurs only for a brief period of life, the need to repair and remodel muscle and bone continues throughout life. Maintaining the health of muscle and bone is an essential part of the aging process and critical to maintain mobility, health and the active tissues of our body. Protein needs become more important during periods of reduced food intake such as weight loss or during periods of recovery after illness or during aging.

### • Protein needs are proportional to body weight; NOT energy intake

Protein needs for adults relate to body weight. Dietary protein need is often presented as a percentage of energy intake. The DRIs represent the acceptable protein range as 10% to 35% of total energy. However, protein needs are constant across all energy intakes. So at low energy intakes, protein needs to be a higher percentage of total calories and at high energy intakes protein can be reduced as a percentage of total calories. In general, dietary protein should be established first in any diet in proportion to body weight and then carbohydrates and fats added determined by energy needs.

### • Optimal adult protein use is a function of intake at individual meals

Protein is an important part of good nutrition at every meal. Vitamins and minerals can fulfill nutrient needs on a once-per-day basis but for protein the body has no ability to store a daily supply. To maintain healthy muscles and bones for adults, at least 30 g of protein should be consumed at more than one meal. Breakfast is an important meal for dietary protein because the body is in a catabolic state after an overnight fast. A meal with at least 30 g of protein is required to initiate repletion of body proteins. Protein at breakfast is also critical for regulation of appetite and daily food intake.

### • Most adults benefit from protein intakes above the minimum RDA

Aging populations confront increasing incidence of obesity, osteoporosis, type 2 diabetes, Metabolic Syndrome, heart disease, and sarcopenia which have raised new questions about dietary ratios of carbohydrates, fats, and protein for life-long health. The RDA represents the minimum daily intake for active healthy adults. For most adults, replacing some dietary carbohydrates with protein will help to maintain body composition and mobility, improve blood lipids and lipoproteins, and help to control food intake.

## Competing interests

DKL has received honorarium for participation in speaker bureaus for the National Dairy Council (NDC) and National Cattlemen's Beef Association (NCBA), serves on the Research Advisory Board for the Egg Nutrition Center (ENC), and has research funding from NDC and ENC.
